# Cerebellar control of gait and interlimb coordination

**DOI:** 10.1007/s00429-014-0870-1

**Published:** 2014-08-20

**Authors:** María Fernanda Vinueza Veloz, Kuikui Zhou, Laurens W. J. Bosman, Jan-Willem Potters, Mario Negrello, Robert M. Seepers, Christos Strydis, Sebastiaan K. E. Koekkoek, Chris I. De Zeeuw

**Affiliations:** 1 Department of Neuroscience, Erasmus MC, P.O. Box 2040, 3000 CA Rotterdam, The Netherlands; 2 Netherlands Institute for Neuroscience, Royal Academy of Arts and Sciences, 1105 BA Amsterdam, The Netherlands

**Keywords:** Purkinje cells, Interneurons, Granule cells, Locomotion, Interlimb coordination, Erasmus Ladder

## Abstract

Synaptic and intrinsic processing in Purkinje cells, interneurons and granule cells of the cerebellar cortex have been shown to underlie various relatively simple, single-joint, reflex types of motor learning, including eyeblink conditioning and adaptation of the vestibulo-ocular reflex. However, to what extent these processes contribute to more complex, multi-joint motor behaviors, such as locomotion performance and adaptation during obstacle crossing, is not well understood. Here, we investigated these functions using the Erasmus Ladder in cell-specific mouse mutant lines that suffer from impaired Purkinje cell output (Pcd), Purkinje cell potentiation (L7-Pp2b), molecular layer interneuron output (L7-Δγ2), and granule cell output (α6-Cacna1a). We found that locomotion performance was severely impaired with small steps and long step times in Pcd and L7-Pp2b mice, whereas it was mildly altered in L7-Δγ2 and not significantly affected in α6-Cacna1a mice. Locomotion adaptation triggered by pairing obstacle appearances with preceding tones at fixed time intervals was impaired in all four mouse lines, in that they all showed inaccurate and inconsistent adaptive walking patterns. Furthermore, all mutants exhibited altered front–hind and left–right interlimb coordination during both performance and adaptation, and inconsistent walking stepping patterns while crossing obstacles. Instead, motivation and avoidance behavior were not compromised in any of the mutants during the Erasmus Ladder task. Our findings indicate that cell type-specific abnormalities in cerebellar microcircuitry can translate into pronounced impairments in locomotion performance and adaptation as well as interlimb coordination, highlighting the general role of the cerebellar cortex in spatiotemporal control of complex multi-joint movements.

**Electronic supplementary material:**

The online version of this article (doi:10.1007/s00429-014-0870-1) contains supplementary material, which is available to authorized users.

## Introduction

An intact cerebellum is essential for on-the-fly corrections of posture and gait (Armstrong 1986; Morton and Bastian 2006; Morton et al. 2004). Accordingly, a typical sign of cerebellar dysfunction is gait ataxia, which is characterized by balance problems and walking abnormalities (Holmes 1917; Ferrarin et al. 2005). In the clinic, ataxia is often seen after structural cerebellar damage, such as following stroke, paraneoplastic syndromes or genetic mutations (Coesmans et al. 2003; Ilg et al. 2008; De Zeeuw et al. 2011). From a functional modeling perspective, ataxic gait can be interpreted as a failure of the cerebellum to develop an implicit representation of the external world and/or predict consequences of motor commands (Blakemore et al. 2001; Bastian 2006; Shadmehr and Krakauer 2008; Franklin and Wolpert 2011).

Even though gait ataxia and limb coordination have been investigated thoroughly in human subjects, their cellular underpinnings have been relatively neglected due to technical deficiencies in measuring all assets of locomotion in mice. So far, systematic studies on cellular functions in cerebellar motor control have been mainly restricted to adaptive reflex movements around single joints, such as eyeblink conditioning and adaptation of the vestibulo-ocular reflex (VOR), which can be readily measured in mouse mutants (De Zeeuw and Yeo 2005). Indeed, synaptic and intrinsic processing in cerebellar Purkinje cells, interneurons and granule cells all have been shown to underlie particular, often overlapping, aspects of such motor behaviors (Wulff et al. 2009; Galliano et al. 2013a; Schonewille et al. 2010). For example, Purkinje cell potentiation and interneuron inhibition are relevant for both VOR performance and adaptation (Schonewille et al. 2010), whereas the bulk of granule cells are predominantly relevant for VOR adaptation only (Galliano et al. 2013a). Instead, the specific contributions of these cellular functions to more complex, multi-joint and multi-organ motor functions, such as posture and gait, are unclear. To date, it remains to be elucidated whether the various functions of specific cerebellar cell types mentioned above play a critical role in locomotion performance and adaptation (Schonewille et al. 2011), and in particular in interlimb coordination (Zhou et al. 2014).

**Fig. 1:**
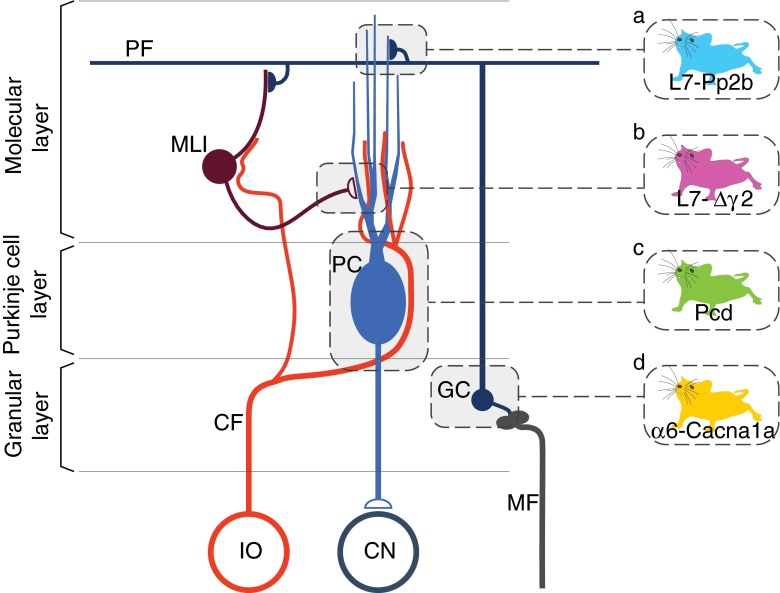
Microcircuitry of the cerebellar cortex highlighting the main sites affected in the Pcd, L7-Pp2b, L7-∆γ2 and α6-Cacna1a mice. The two main excitatory afferents of the cerebellar cortex are the mossy fibers (MF) and climbing fibers (CF). Whereas the MFs originate from various sources in the brainstem, all CFs are derived from the inferior olive (IO). The CFs directly innervate the Purkinje cells (PCs) and influence via non-synaptic release the activity of molecular layer interneurons (MLI), which inhibit PCs. The MFs directly innervate the granule cells (GCs), which in turn give rise to the parallel fibers (PFs) that innervate both PCs and MLIs. PCs form the sole output of the cerebellar cortex to the cerebellar nuclei (CN). The mutants used in the current study either lack Purkinje cells (Pcd, indicated in *green*), intrinsic Purkinje cell plasticity and parallel fiber-to-Purkinje cell potentiation (L7-Pp2b, *blue*), phasic inhibition provided by molecular layer interneurons (L7-∆γ2, *purple*), or most of their granule cell output (α6-Cacna1a, *yellow*)

Here, we studied such behavioral traits in four cell-specific mutant lines including mice lacking Purkinje cell output (Fernandez-Gonzalez et al. 2002; Mullen et al. 1976); mice lacking parallel fiber-to-Purkinje cell long-term potentiation and Purkinje cell intrinsic plasticity (L7-Pp2b mice) (Schonewille et al. 2011); mice lacking phasic Purkinje cell inhibition (L7-Δγ2 mice) (Wulff et al. 2009); and mice with impaired granule cell output (α6-Cacna1a mice) (Galliano et al. 2013a) (Fig. 1). This collection of cerebellar mouse mutants covers the entire spectrum ranging from degeneration of Purkinje cells severely affecting the sole output of the cerebellar cortex to functional ablation of the output of part of the granule cells subtly manipulating the main input stage of this cortex. To study their locomotion performance and adaptation, as well as interlimb coordination, we used the fully automated Erasmus Ladder, yielding systematic descriptions of locomotion in mice (Vinueza Veloz et al. 2012). Importantly, the Erasmus Ladder triggers locomotion adaptation by pairing obstacle appearances with preceding tones at fixed intervals, and allows measurements of interlimb coordination by independent detection of the step cycle of all four limbs.

## Materials and methods

### Animals

We used four different types of wild-type controls and mutants including Pcd mice, L7-Pp2b mice, L7-Δγ2 mice and α6-Cacna1a mice, all of which had a C57BL/6 background. Pcd mice, which lose virtually all Purkinje cells between post-natal days 15 and 30 due to a spontaneous mutation in the *Nna1* gene (Fernandez-Gonzalez et al. 2002; Mullen et al. 1976), were purchased from the Jackson Laboratory (Bar Harbor ME; stock number 000537). L7-Pp2b mice, which lack the regulatory subunit (CNB1) of calcineurin in their Purkinje cells and therefore show impaired intrinsic plasticity and parallel fiber-to-Purkinje cell long-term potentiation (LTP), while maintaining normal parallel fiber-to-Purkinje cell long-term depression (LTD) (Schonewille et al. 2010), were obtained by crossing mice carrying a floxed *Cbn1* gene with mice from an L7-*Cre* line (Barski et al. 2000; Zeng et al. 2001). L7-Δγ2 mice, which lack the GABA_A_ receptor γ2 subunit in their Purkinje cells and thereby show impaired phasic inhibition induced by molecular layer interneurons (Wulff et al. 2009), were obtained by crossing mice carrying a floxed *Gabrg2* gene with mice from the L7-*Cre* line (Barski et al. 2000; Wulff et al. 2007). Finally, α6-Cacna1a mice, which lack P/Q-type Ca^2+^ channels in >75 % of their granule cells and thereby show a reduced potential for excitation of both Purkinje cells and molecular layer interneurons (Galliano et al. 2013a), were obtained by crossing mice carrying a floxed *Cacna1a* gene with mice having the *Cre* transgene under control of the GABRA6 promoter (Aller et al. 2003; Todorov et al. 2006). In total, 35 mutants (for Pcd *n* = 5 males, L7-Pp2b *n* = 5 males and 7 females, L7-Δγ2 *n* = 4 males and 6 females, and α6-Cacna1a *n* = 5 males and 3 females) and 37 control littermates (*n* = 7 males, *n* = 12 females, *n* = 5 males and 5 females, and *n* = 6 males and 2 females, respectively) were tested on the Erasmus Ladder. For the Pcd mice we used heterozygous littermates as controls, while for the L7-Pp2b mice, L7-Δγ2 mice and α6-Cacna1a mice we used Cre^−^/loxP^+/+^, Cre^−^/loxP^−/−^ and Cre^+^/loxP^−/−^ mice as controls. At the start of the experiment Pcd mice were 4–6 weeks old, i.e., after the occurrence of Purkinje cell degeneration but before other brain regions were affected (O’Gorman and Sidman 1985; Mullen et al. 1976; Zhang et al. 1999). Mice of the other three strains were between 4 and 6 months of age. All mice were kept on a 12 h light/dark cycle, and were healthy, except for the Pcd mutants showing their typical ataxic phenotype. All mice had free access to standard laboratory food and water showing a regular weight corresponding to their age and genotype (see also Mullen et al. 1976; O’Gorman and Sidman 1985; Zhang et al. 1999; Wulff et al. 2009; Schonewille et al. 2010; Galliano et al. 2013a, b). All experiments were approved by the institutional Animal Welfare Board as required by Dutch and EU legislation and guidelines.

### Equipment and behavioral protocol

**Fig. 2:**
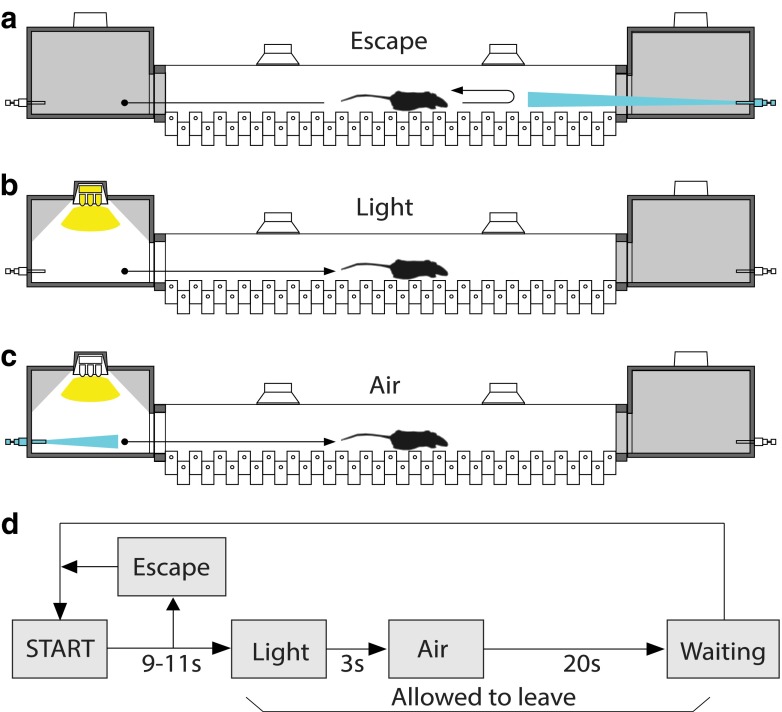
The Erasmus Ladder test. The Erasmus Ladder consists of a *horizontal* ladder situated between two shelter boxes. The sequence of illustrations shows how the paradigm works. **a** The mouse has to stay inside the dark shelter during a random time interval that varies between 9 and 11 s before it is allowed to walk on the ladder. Whenever the mouse tries to cross the ladder before the time interval has passed, a powerful crosswind coming from the opposite shelter is activated, pushing the mouse back to its starting position; we refer to such a trial as an “escape” trial. **b** When the time interval has passed, the LED light in the roof turns on (“light”) and the mouse is allowed to leave the shelter box. The light remains on until the mouse reaches the opposite shelter. **c** If the mouse does not leave the shelter within 3 s after the light goes on, a powerful air puff from the back of the shelter is activated (“air”). Normally, this stimulus is enough to encourage the mouse to start walking on the ladder. **d** Schematic representation of the temporal order of the events mentioned above

**Fig. 3:**
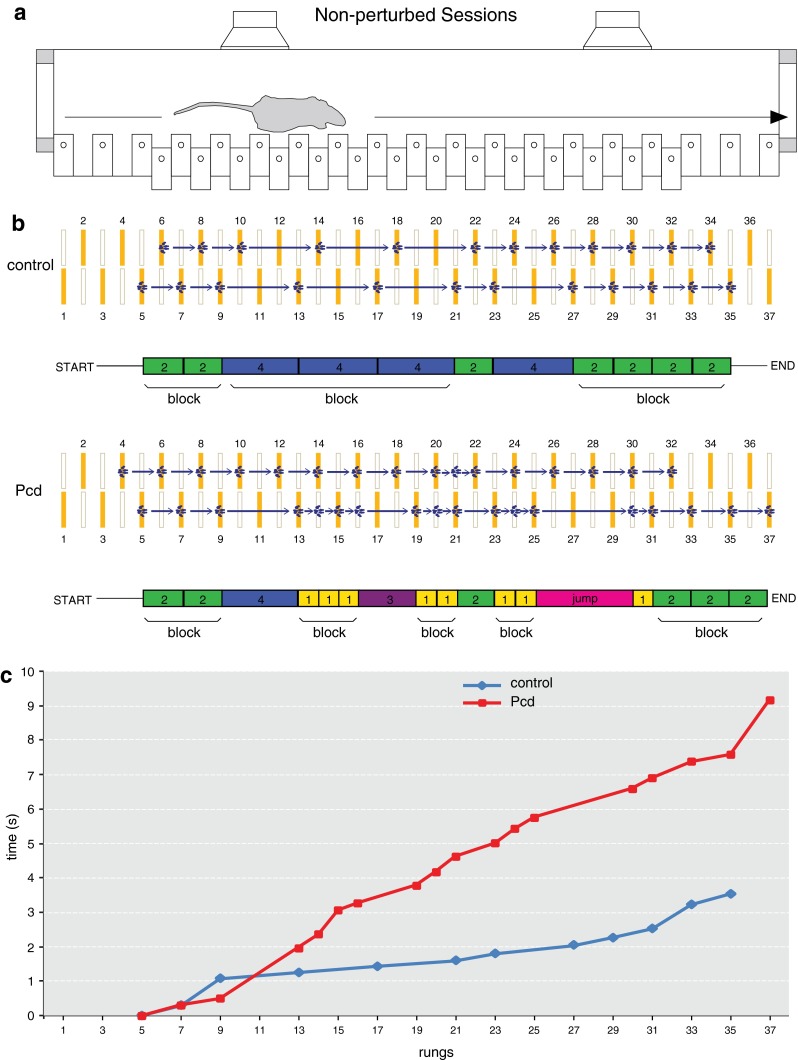
*Baseline* locomotion is tested during non-perturbed sessions. **a** Each daily session consisted of 72 trials, during which the mice had to walk back and forth from one shelter box to the other. Right from the beginning of the experiment, most of the mice usually stepped only on the upper rungs and only infrequently touched the lower ones, which was considered as a misstep. **b** The rungs of the ladder have custom-made pressure sensors. The upper rungs, which are indicated by *closed yellow symbols*, are positioned in a left–right alternating pattern. The *blue* footprints represent the typical touches of the front paws of a control (*top*) and Pcd mouse (*bottom*) during a representative trial on the ladder. A single step (*arrow*) corresponds to a front paw step. The steps are classified according to their length and direction, and they are represented as *colored rectangles* located below the ladders. Consecutive single steps of the same length merge to build “blocks”. **c** Time course of the trials is depicted in **b**. *Symbols* represent single touches

To study locomotion and cognitive capabilities in mice, we used the fully automated Erasmus Ladder. Details on the device and its software have been published (Van Der Giessen et al. 2008; Vinueza Veloz et al. 2012). In short, the Erasmus Ladder consists of a horizontal ladder between two shelter boxes, each equipped with an LED spotlight in the roof and two pressurized air outlets in the back. Sensory stimuli (light and air) serve to control the moment of departure of the mice (Fig. 2). The ladder itself has 37 rungs on each side, and each rung can be displaced vertically following a command from the control system. Even-numbered rungs on one side and odd-numbered rungs on the other were elevated by 6 mm, thereby creating a left/right alternating pattern (Fig. 3). All rungs are equipped with custom-made pressure sensors that are continuously monitored. The setup is controlled by software written in LabView (National Instruments, Austin, TX, USA) that operates with a fixed cycle of 2 ms.

**Fig. 4:**
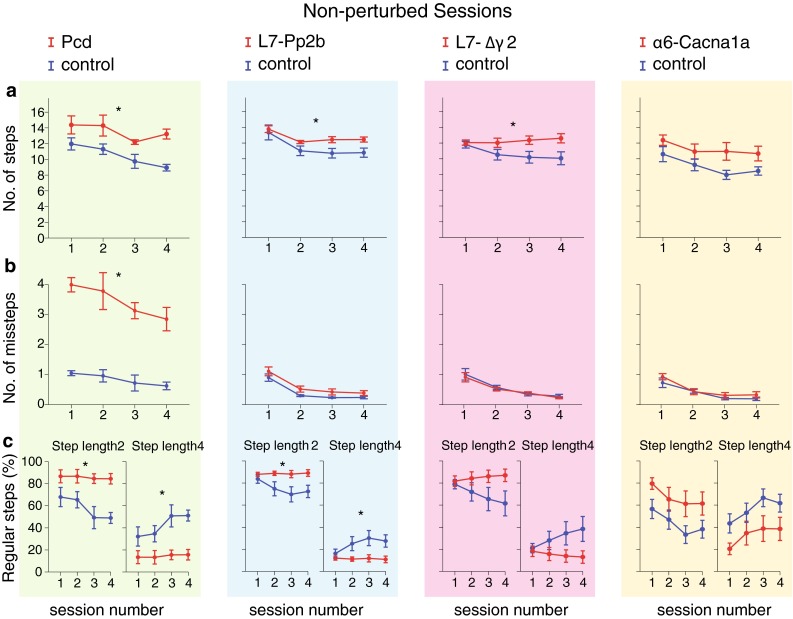
Non-perturbed locomotion: number of steps, missteps and distribution of step sizes. **a** Most cerebellar mutant mice (Pcd *n* = 5; L7-Pp2b *n* = 12; L7-Δγ2 *n* = 10; α6-Cacna1a *n* = 8) used significantly more steps to cross the Erasmus Ladder than controls (Pcd control *n* = 7; L7-Pp2b control *n* = 12; L7-Δγ2 control *n* = 10; α6-Cacna1a control *n* = 8). **b** Accuracy was tested by estimating the average number of missteps per trial. Only Pcd mice showed an abnormally high number of missteps in comparison to control mice. **c** Distribution of step sizes was tested by quantifying the occurrence of small (step length = 2) and large regular steps (step length = 4). Both Pcd and L7-Pp2b mice had a significantly higher rate of small steps and a significantly lower rate of large steps than control littermates. *Error bars* represent SEM. Significant differences between mutant and control mice are indicated with *asterisks*

**Fig. 5:**
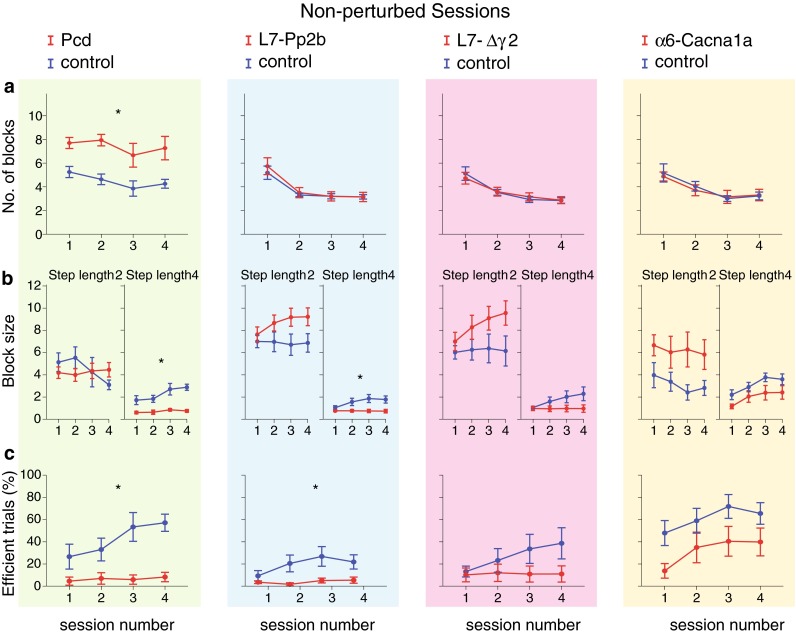
Non-perturbed locomotion: walking pattern consistency and efficiency. **a** To estimate the consistency of the walking pattern, we calculated the mean number of blocks with steps of the same length for each trial (see Fig. 3b). Only Pcd mice changed their step lengths significantly more often than control mice. **b** Although some non-significant trends emerged, all cerebellar mutant mice showed a similar number of consecutive small steps (i.e., block size for small steps) compared to control mice. In contrast, with respect to large steps Pcd and L7-Pp2b mice made significantly fewer consecutive steps, keeping the average block size small. **c** To estimate the efficiency of their walking patterns, we calculated the percentage of trials per session, in which the maximum number of large steps or leaps was higher than that of the other steps (efficient trials). Pcd and L7-Pp2b mice showed a significantly lower rate of efficient trials per session, while L7-Δγ2 and α6-Cacna1a mice showed a trend that did not reach significance. *Error bars* represent SEM. Significant differences between mutant and control mice are indicated with *asterisks*

**Fig. 6:**
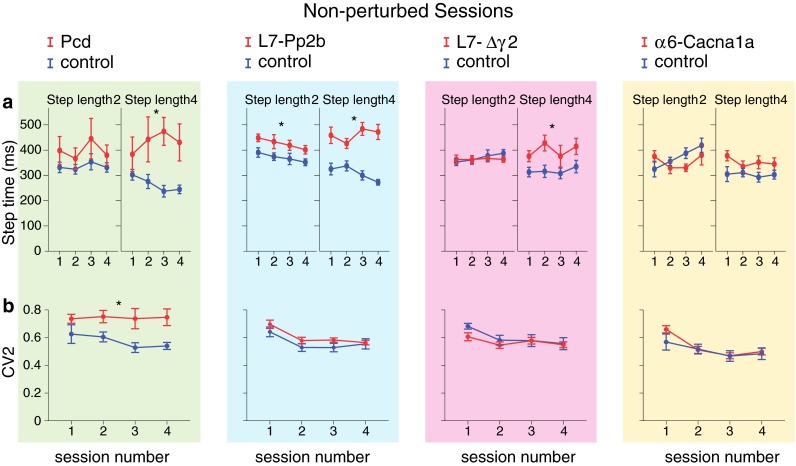
Non-perturbed locomotion: temporal control. **a** Step time corresponds to the elapsed time (in ms) between two consecutive touches (see Fig. 3). For small steps only L7-Pp2b mice had significantly longer step times than control mice, whereas for large steps this held true not only for L7-Pp2b, but also for Pcd and L7-Δγ2 mice. **b** The variability of step times (CV2) was only significantly higher for Pcd mice with respect to that in controls. *Error bars* represent SEM. Significant differences between mutant and control mice are indicated with *asterisks*

**Fig. 7:**
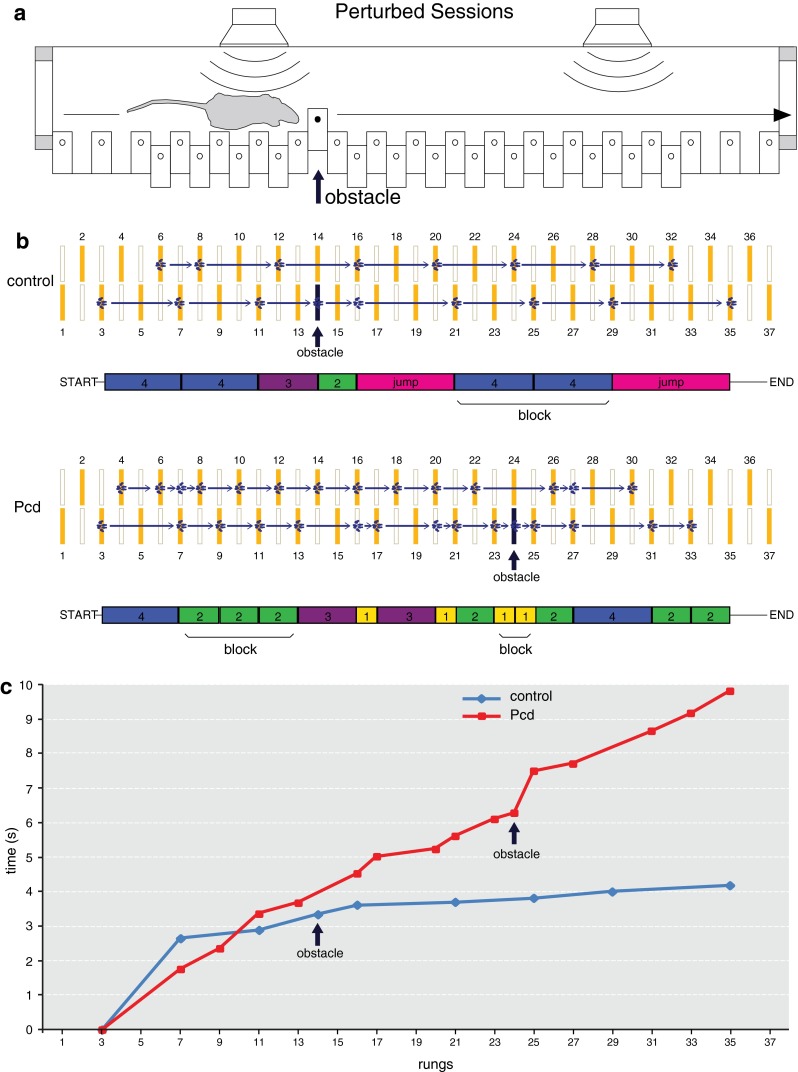
Locomotion adaptation is tested during perturbed sessions. **a** Throughout the perturbed sessions, the mice learned to adapt their walking patterns in response to a 15 kHz auditory stimulus preceding the appearance of an obstacle in their pathway. The obstacle, which consisted of an upward moving rung, was always located on the right side of the mouse independently of its walking direction. Its specific location depended on the predicted position of the mouse on the ladder, but was otherwise randomized. **b** The *blue* footprints represent the front paw touches of the same control and Pcd mice depicted in Fig. 3, but now during a perturbed trial. The position of the obstacle is indicated with *black arrows*. **c** Time course of the trials is depicted in **b**. *Symbols* represent single touches

**Fig. 8:**
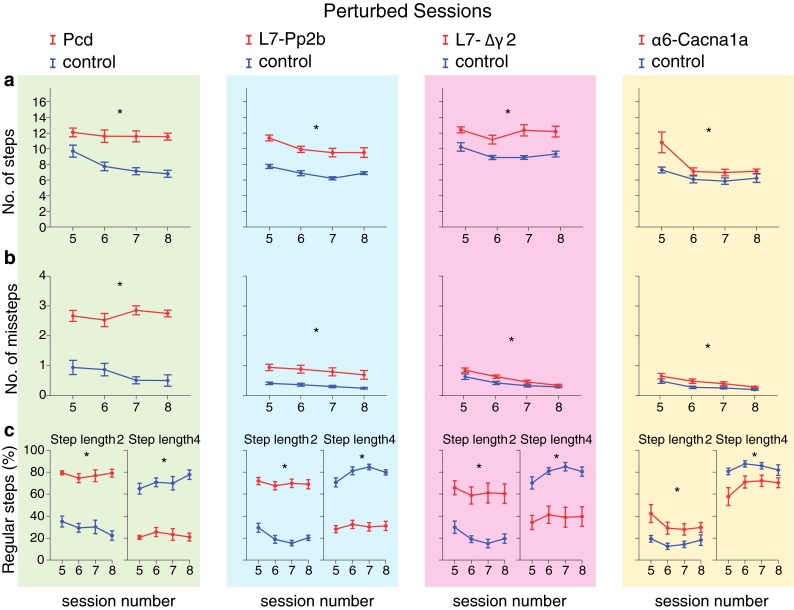
Locomotion adaptation: number of steps, missteps and distribution of step sizes. Perturbed sessions are more challenging for mice than non-perturbed sessions. Consequently, throughout these sessions all cerebellar mutant mice showed impairments, some of which were not obvious during the non-perturbed sessions. **a** During perturbed sessions, all cerebellar mutant mice used significantly more steps to cross the ladder than control mice. **b** Likewise, all cerebellar mutant mice showed significantly more missteps. **c** Moreover, all cerebellar mutant mice also showed a significantly higher rate of small steps and a significantly lower rate of large steps. *Error bars* represent SEM. Significant differences between mutant and control mice are demonstrated with an *asterisk*

**Fig. 9:**
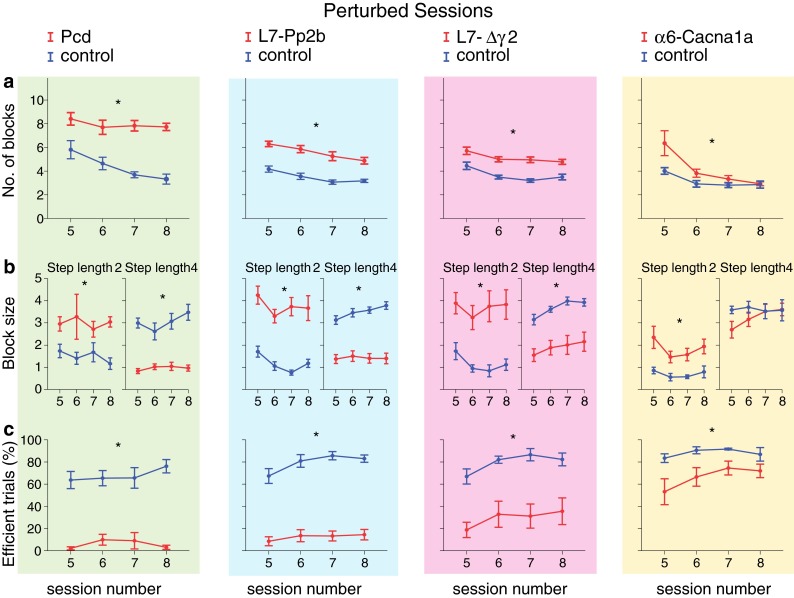
Locomotion adaptation: walking pattern consistency and efficiency. **a** All cerebellar mutant mice showed very inconsistent walking patterns in comparison with control mice throughout the perturbed sessions; mutant mice changed their step lengths significantly more often than control mice. **b** All cerebellar mutant mice showed a significantly higher number of consecutive small steps than control mice. Similarly, except for α6-Cacna1a, cerebellar mutant mice showed a lower number of consecutive large steps, i.e., smaller block sizes. **c** All cerebellar mutant mice had less efficient trials per session than control littermates. *Error bars* represent SEM. Significant differences between mutant and control mice are demonstrated with an *asterisk*

**Fig. 10:**
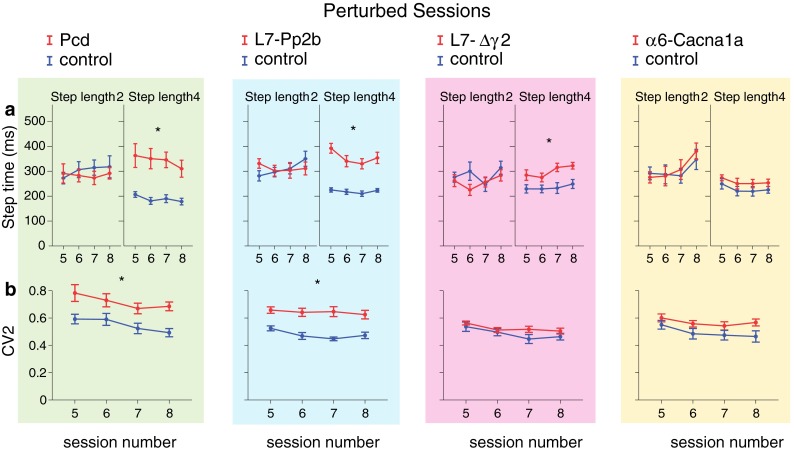
Locomotion adaptation: timing and variability. **a** All cerebellar mutants took a similar amount of time to make a single small step compared to control mice. The opposite occurred with regard to large steps; except for α6-Cacna1a, all cerebellar mutants took more time per step than controls. **b** Only Pcd and L7-Pp2b mice showed an increased variability of their step times in comparison with controls. *Error bars* represent SEM. Significant differences between mutant and control mice are demonstrated with an *asterisk*

For the current study, we followed a paradigm similar to that of a previous study (Vinueza Veloz et al. 2012). Briefly, each mouse had to perform one daily session during 8 days, with 2 days of rest in the middle (i.e., between sessions 4 and 5). Each daily session consisted of 72 trials during which the mouse had to walk back and forth between two shelter boxes. During the first four sessions, we assessed naive locomotion. In these sessions, none of the rungs moved (“non-perturbed sessions”) (Figs. 3, 4, 5, 6). During the last four sessions (i.e., sessions 5–8), we tested locomotion adaptation by challenging the mouse to deal with the appearance of an obstacle, which was preceded by a tone 200 ms prior to its occurrence (“perturbed sessions”) (Figs. 7, 8, 9, 10). The obstacle was induced by elevating one of the lower rungs by 18 mm, thus creating an obstacle of 12 mm just in front of the mouse. The location of the obstacle on the ladder varied randomly between trials, but it always appeared on the right side (independently of the walking direction). The exact timing of the obstacle appearance depended on the walking pattern and the predicted trajectory of the mouse (for details see Van der Giessen et al. 2008). Steps were recorded as touches on the rungs; to prevent false positives, we took into account only touches that lasted >30 ms. To avoid detecting hind limb touches as backward steps, we accepted only sequences of two or more consecutive backward steps as true backward movements. The analyses of forward steps revealed that mice usually step from one elevated rung to the next, skipping the lower rung (i.e., step length = 2), or to the consecutive elevated rung, skipping three rungs (i.e., step length = 4) (see Figs. 3b, 7b). Hence, we considered steps with a step length equal to 2 or 4 to be “regular steps”. Other step lengths, including missteps (i.e., stepping from or to a lower rung), leaps (i.e., step lengths >4) as well as backward steps, occurred less frequently and were therefore termed “irregular steps” (Figs. 3b, 7b). For the analyses of both the unperturbed walking patterns and locomotion adaptation, we only took the right front limb into consideration, since the obstacle was presented only on this side of the ladder. Instead, for the coordination parameters, we used data from all four limbs. To reduce the potential impact of a putative bias due to the air and/or light stimuli in the shelter box, the first and last step of each trial (i.e., stepping out of and into the shelter boxes) were omitted from analyses.

Two cognitive functions were tested with the Erasmus Ladder: motivation and avoidance behavior. The assessment of these cognitive abilities depended on the ability of the mouse to use sensory stimuli (light and air) as indications to initiate walking on the ladder. The trial started when a mouse was positioned inside the starting shelter box. The mouse had to remain inside it for a random period of time (between 9 and 11 s). Whenever the mouse escaped before the time had elapsed, a strong head wind (coming from the shelter box at the opposite end) forced the mouse to go back (Fig. 2a). Once the random time had elapsed, the LED in the starting shelter box was turned on, indicating that the mouse had to leave the shelter box (Fig. 2b). If the mouse did not leave within 3 s after the light was turned on, a strong tailwind forced the mouse to begin walking on the ladder (second cue of departure) (Fig. 2c). When the mouse reached the shelter box at the opposite end, the light and air were turned off and a new cycle started. A schematic description of the possible outcomes and their interactions over time is depicted in Fig. 2d. The variables used to assess motivation and avoidance behavior were the percentages of trials during which a mouse used/needed either light or strong tailwind stimuli to initiate walking on the ladder.

### Data processing

Data collected from the Erasmus Ladder were stored in a relational database (MySQL, Oracle, Redwood Shores, CA, USA) and then processed off-line using custom-written software in LabView and Python (Python Software Foundation, Beaverton, OR, USA). Step lengths were determined by the distance between two consecutive touches. Likewise, step time was defined as the time that elapsed between the onsets of two consecutive touches (Figs. 3c, 7c). The coefficient of variance of adjacent step times (CV2) was calculated as $2 \times \frac{{\left| {{\text{step}}_{n + 1}\, -\, {\text{step}}_{n} } \right|}}{{({\text{step}}_{n + 1}\, +\, {\text{step}}_{n} )}}$. The regularity of stepping patterns was also evaluated by considering “blocks” of consecutive steps with the same step length (Fig. 3b). The length of a block was the number of consecutive steps with the same step length.

For the analyses of interlimb coordination, the “front–hind time” was defined as the time in milliseconds that elapsed between the onset of the front limb touch and the moment when the hind limb on the ipsilateral side released the previous sensor; the “front–hind time” could not be calculated reliably by using the onset-to-onset times, because the hind limb often touched the same sensor as the ipsilateral front limb and hence both touches often temporarily overlapped. The “left–right time” was defined as the time that elapsed between the onset of one front limb touch and the onset of the next front limb touch on the contralateral side. For both front–hind times and left–right times, we only evaluated steps with step lengths of 2 or 4.

### Statistical analyses

Except for the cluster analysis (see below), data were analyzed using SPSS (IBM Corporation, Armonk, NY, USA) and all *p* values were calculated by comparing cerebellar mutant mice with their control littermates. We tested for significant differences between sessions in naive walking patterns, locomotion adaptation, as well as cognition parameters, using two-way repeated measures ANOVA. Improvement within sessions was evaluated using linear regression. For the analysis of interlimb coordination, we used Matlab (MathWorks, Natick MA) to run two-dimensional Kolmogorov–Smirnov tests (2-D Kolmogorov–Smirnov test).

Cluster analysis was performed using PAST software (Hammer et al. 2001). First, we normalized all quantifiable parameters of session 5 (average number of steps per trial, average number of missteps per trial, average ratio between steps with step lengths 2 and 4, average block size of step lengths 2 and 4, average number of efficient trials, average step time of step lengths 2 and 4 and average step time CV2) to values between 0 and 1. Next, we performed a principal component analysis and a cluster analysis using Ward’s method (with 1,000 bootstraps).

## Results

To study unperturbed locomotion patterns, locomotion adaptation when crossing obstacles, interlimb coordination as well as cognitive parameters of motivation and avoidance, we subjected four different cell type-specific mutant mouse lines including Pcd, L7-Pp2b mice, L7-Δγ2 mice and α6-Cacna1a mice to the Erasmus Ladder task.

### Naive locomotion

#### Number of steps, accuracy and step length

We first tested naive locomotion during four non-perturbed sessions. Throughout these sessions, the mice had to walk from a shelter box on one side to the shelter box on the opposite side, and vice versa, 72 times every day during a 4-day period (Fig. 3). Most mutant mice used more steps than their control littermates to travel from one box to the other (Pcd *F*_(1,10)_ = 13.82, *p* = 0.004; L7-Pp2b *F*_(1,22)_ = 4.67, *p* = 0.042; L7-Δγ2 *F*_(1,18)_ = 5.08, *p* = 0.037; α6-Cacna1a *F*_(1,14)_ = 4.32, *p* = 0.056) (Fig. 4a). Most of these steps (>80 %) were regular steps from one elevated rung to the next elevated rung (see “Materials and methods”). Only Pcd mice had an abnormally high percentage (approximately, 40 %) of irregular steps (data not shown). Similarly, only Pcd mice made more missteps than control mice (Pcd *F*_(1,10)_ = 166.12, *p* < 0.001; L7-Pp2b *F*_(1,22)_ = 3.30, *p* = 0.083; L7-Δγ2 *F*_(1,18)_ = 1.93, *p* = 0.665; α6-Cacna1a *F*_(1,14)_ = 1.02, *p* = 0.331) (Fig. 4b). We next compared the occurrence of small regular steps (step length = 2) with that of large regular steps (step length = 4). All cerebellar mutants made on average fewer large regular steps than the control littermates, but this difference was not statistically significant for the L7-Δγ2 and α6-Cacna1a mice (Pcd *F*_(1,10)_ = 7.19, *p* = 0.023; L7-Ppp2b *F*_(1,22)_ = 5.62, *p* = 0.027; L7-∆γ2 *F*_(1,18)_ = 2.44, *p* = 0.136; α6-Cacna1a *F*_(1,14)_ = 3.88, *p* = 0.069) (Fig. 4c). Over the course of the sessions, virtually all controls gradually increased the number of large steps at the expense of small ones (for *p* values, see Table 1). In contrast, the occurrence of large or small steps remained unaltered over the sessions in the Pcd, L7-Pp2b and L7-Δγ2 mice. Only in the α6-Cacna1a mice we observed a gradual increase in the occurrence of large steps (for *p* values, see Table 1).

**Table 1:** Locomotion parameters change over time

Parameters	Control *p*	Pcd *p*	Control *p*	L7-Pp2b *p*	Control *p*	L7-∆y2 *p*	Control *p*	α6-Cacna1a *p*
Non-perturbed								
No. of steps	*0.005*	0.310	*0.026*	*0.017*	0.078	0.438	*0.008*	*0.027*
No. of missteps	0.141	0.159	*0.000*	*0.000*	0.003	*0.003*	*0.009*	*0.000*
Regular steps (%)								
Step length 2	*0.028*	0.570	*0.022*	0.900	0.072	0.291	*0.003*	*0.037*
Step length 4	*0.028*	0.570	*0.022*	0.900	0.072	0.291	*0.003*	*0.037*
No. of blocks	0.110	0.503	*0.005*	*0.000*	0.003	*0.000*	*0.044*	*0.000*
Block size								
Step length 2	0.060	0.504	0.972	*0.000*	0.854	*0.007*	0.173	0.703
Step length 4	*0.024*	0.140	*0.021*	0.959	0.053	0.944	*0.001*	*0.015*
Efficient trials (%)	*0.012*	0.169	*0.038*	0.337	0.047	0.812	*0.029*	*0.011*
Step time (ms)								
Step length 2	0.784	0.313	0.273	0.051	0.317	0.943	0.062	0.374
Step length 4	0.173	0.561	*0.001*	0.143	0.523	0.256	0.861	0.427
CV2	0.174	0.995	*0.015*	*0.000*	0.003	*0.039*	0.099	*0.000*
Perturbed								
No. of steps	*0.030*	0.740	*0.000*	*0.001*	0.028	0.083	*0.021*	*0.017*
No. of missteps	*0.017*	0.310	*0.001*	*0.001*	0.002	*0.000*	*0.000*	*0.000*
Regular steps (%)								
Step length 2	0.277	0.370	*0.006*	0.609	0.019	0.225	0.087	0.051
Step length 4	0.277	0.370	*0.006*	0.609	0.019	0.225	0.087	0.051
No. of blocks	*0.001*	0.413	*0.000*	*0.000*	0.000	*0.012*	*0.001*	*0.015*
Block size								
Step length 2	0.464	0.854	*0.001*	0.153	0.050	0.149	0.087	0.100
Step length 4	0.066	0.730	*0.002*	0.698	0.000	*0.036*	0.902	*0.003*
Efficient trials (%)	0.572	0.438	*0.016*	0.197	0.038	0.054	0.346	0.068
Step time (ms)								
Step length 2	0.396	0.819	0.069	0.114	0.152	*0.008*	0.439	*0.036*
Step length 4	0.192	0.285	0.276	*0.001*	0.131	*0.002*	0.051	0.329
CV2	0.078	0.142	*0.010*	0.470	0.000	*0.000*	*0.012*	0.135

With the exception of Pcd mice, all the cerebellar mutant mouse lines exhibited changes in their locomotion parameters over the course of the sessions. The *p* values for repeated measures ANOVA, separated into non-perturbed (1–4) and perturbed (5 to 8) sessions are indicated. Significant differences (*p* < 0.05) are indicated in italics

Next, we evaluated whether changes in motor performance could also be observed within sessions, comparing controls with the mutants with the most prominent phenotype, i.e., Pcd mice. We plotted the number of steps, number of missteps and the variability in timing of consecutive steps (CV2; see “Materials and methods”) and performed linear regression analysis. Whereas we could not find a significant improvement for any of these parameters in Pcd mice, we found several in control mice (Suppl. Figure 1 and Suppl. Table 1). These improvements occurred mainly during the first session (number of steps *p* > 0.001; number of missteps *p* = 0.002; CV2 *p* = 0.001; cf. Pcd mice: number of steps *p* = 0.968; number of missteps *p* = 0.566; CV2 *p* = 0.968). Thus, in control mice improvement occurred not only across, but also within sessions.

#### Walking pattern consistency and efficiency

To analyze the consistency of their walking patterns, we investigated how frequently the mice changed their step length. We identified blocks of consecutive steps with the same length and then calculated the average number of such blocks per trial as well as the maximum number of steps per block (Fig. 5). During the first session, control mice changed their step lengths multiple times (approximately 6 times). Only Pcd mice changed their step lengths significantly more often than their littermates (Pcd *F*_(1,10)_ = 35.27, *p* < 0.001; L7-Ppp2b *F*_(1,22)_ = 0.14, *p* = 0.714; L7-∆γ2 *F*_(1,18)_ = 0.00, *p* = 0.966; α6-Cacna1a *F*_(1,14)_ = 0.04, *p* = 0.841) (Fig. 5a). As training progressed, all groups made fewer changes in their step lengths. The only exception was Pcd mice, which kept walking irregularly (for *p* values, see Table 1).

Elaborating on the finding that cerebellar mutants made more steps per trial than controls, we calculated the number of regular steps per block (see “Materials and methods”). Unlike the block sizes for small regular steps (step length = 2), for which we found no statistically significant difference between mutants and controls (Pcd *F*_(1,10)_ = 0.812, *p* = 0.389; L7-Pp2b *F*_(1,22)_ = 3.10, *p* = 0.092; L7-∆γ2 *F*_(1,18)_ = 2.66, *p* = 0.120; α6-Cacna1a *F*_(1,14)_ = 4.36, *p* = 0.056), those for large regular steps (step length = 4) were significantly smaller in Pcd and L7-Pp2b mice (Pcd *F*_(1,10)_ = 16.51, *p* = 0.002; L7-Pp2b *F*_(1,22)_ = 7.70, *p* = 0.011; L7-∆γ2 *F*_(1,18)_ = 2.70, *p* = 0.118; α6-Cacna1a *F*_(1,14)_ = 3.45, *p* = 0.086) (Table 2; Fig. 5b). Since most controls progressively increased the rate of large steps over the course of session one to four (Fig. 5b), it is likely that by increasing the length of their steps, they improved their efficiency when walking on the ladder. To measure the level of efficiency, we calculated the percentage of trials per session in which the maximum number of consecutive large steps or jumps was higher than that of the other steps (Fig. 5c). All control groups as well as the α6-Cacna1a mutants, but not the Pcd, L7-Pp2b and L7-∆γ2 mutants, improved their efficiency with training (for *p* values, see Table 1). Moreover, Pcd and L7-Pp2b mice, but not L7-∆γ2 and α6-Cacna1a, had an overall significantly lower rate of efficient trials per session than control littermates (Pcd *F*_(1,10)_ = 7.51, *p* = 0.021; L7-Pp2b *F*_(1,22)_ = 6.33, *p* = 0.020; L7-∆γ2 *F*_(1,18)_ = 1.76, *p* = 0.201; α6-Cacna1a *F*_(1,14)_ = 3.93, *p* = 0.068) (Fig. 5c).

**Table 2:** Behavior of cerebellar mutant mice during non-perturbed sessions

Locomotion performance												Interlimb coordination		Motivation
Mouse line	No. of steps	No. of missteps	Regular steps (%)		No. of blocks	Block size		Effc. trials (%)	Step time (ms)		CV2			
			SL 2	SL 4		SL 2	SL 4		SL 2	SL 4		Front–hind	Left–right	
Pcd	↑	↑	↑	↓	↑	n	↓	↓	n	↑	↑	imp.	imp.	n
L7-Pp2b	↑	n	↑	↓	n	n	↓	↓	↑	↑	n	imp.	imp.	n
L7-∆y2	↑	n	n	n	n	n	n	n	n	↑	n	imp.	imp.	n
α6-Cacna1a	n	n	n	n	n	n	n	n	n	n	n	imp.	imp.	n

During the non-perturbed sessions locomotion performance was severely impaired in Pcd and L7-Pp2 mice, but only slightly altered in L7-Δγ2 and α6-Cacna1a mice. At the same time, all four cerebellar mutant mouse lines exhibited poor interlimb coordination. None of the cerebellar mouse lines displayed deficits in motivation. Significant increases (↑) and decreases (↓) relative to control littermates are indicated; n indicates no significant difference found with repeated measures ANOVA

*SL 2* step length = 2, *SL 4* step length = 4, *Effc. trials* efficient trials, *imp.* impaired

#### Temporal aspects of locomotion

Mutant mice needed more time to make a step. This was especially obvious for large regular steps (step length = 4: Pcd *F*_(1,8)_ = 28.92, *p* = 0.001; L7-Pp2b *F*_(1,22)_ = 29.84, *p* < 0.001; L7-∆γ2 *F*_(1,18)_ = 6.38, *p* = 0.021; α6-Cacna1a *F*_(1,14)_ = 4.45, *p* = 0.053), but not so much for small regular steps (step length = 2: Pcd *F*_(1,10)_ = 1.44, *p* = 0.258; L7-Pp2b *F*_(1,22)_ = 6.31, *p* = 0.020; L7-∆γ2 *F*_(1,18)_ = 0.12, *p* = 0.730; α6-Cacna1a *F*_(1,14)_ = 1.09, *p* = 0.314) (Fig. 6a). The CV2 was significantly larger in Pcd mice than their control littermates, whereas the other mutant lines showed a CV2 for step time comparable to that in controls (Pcd *F*_(1,10)_ = 11.11, *p* = 0.048; L7-Pp2b *F*_(1,22)_ = 1.88, *p* = 0.185; L7-∆γ2 *F*_(1,18)_ = 0.67, *p* = 0.424; α6-Cacana1a *F*_(1,14)_ = 0.45, *p* = 0.514) (Fig. 6b).

### Locomotion adaptation

Next, we determined whether the cerebellar mutants were able to adapt their walking patterns to environmental changes. To this end we subjected all mice to four consecutive “perturbed sessions”, during which they learned on their route from one box to the other to adapt their walking patterns to an auditory stimulus preceding the appearance of an obstacle (see “Materials and methods” and Fig. 7). In general, the cerebellar mutants, in particular the L7-∆γ2 and α6-Cacna1a mice, showed several significant impairments during locomotion adaptation that were not obvious during non-perturbed sessions (compare Tables 2 and 3).

**Table 3:** Behavior of cerebellar mutant mice during perturbed sessions

Locomotion adaptation												Interlimb coordination		Avoidance behavior
Mouse line	No. of steps	No. of missteps	Regular steps (%)		No. of blocks	Block size		Effc. trials (%)	Step time (ms)		CV2			
			SL 2	SL 4		SL 2	SL 4		SL 2	SL 4		Front–hind	Left–right	
Pcd	↑	↑	↑	↓	↑	↑	↓	↑	n	↓	↓	imp.	imp.	n
L7-Pp2b	↑	↑	↑	↓	↑	↑	↓	↑	n	↓	↓	imp.	imp.	n
L7-∆y2	↑	↑	↑	↓	↑	↑	↓	↑	n	↓	n	imp.	imp.	n
α6-Cacna1a	↑	↑	↑	↓	↑	↑	n	↑	n	n	n	imp.	imp.	n

During perturbed sessions locomotion adaptation and interlimb coordination were severely impaired in all four cerebellar mouse mutant lines. None of the cerebellar mouse lines displayed deficits in avoidance behavior. Significant increases (↑) and decreases (↓) relative to control littermates are indicated; n indicates no significant difference tested with repeated measures ANOVA

*SL 2* step length = 2, *SL 4* step length = 4, *Effc. trials* efficient trials, *imp.* impaired

#### Number of steps, accuracy and step length

Even more than in non-perturbed sessions, during the course of perturbed sessions all cerebellar mutant mice used considerably more steps than their control littermates (Pcd *F*_(1,10)_ = 47.46, *p* < 0.001; L7-Pp2b *F*_(1,22)_ = 54.9, *p* < 0.001; L7-∆γ2 *F*_(1,18)_ = 19.98, *p* < 0.001; α6-Cacna1a *F*_(1,14)_ = 7.3, *p* = 0.018) (Fig. 8a). Similarly, all cerebellar mutant mice made significantly more missteps than controls (Pcd *F*_(1,10)_ = 68.94, *p* < 0.001; L7-Pp2b *F*_(1,22)_ = 14.61, *p* = 0.001; L7-∆γ2 *F*_(1,18)_ = 5.79, *p* = 0.027; α6-Cacna1a *F*_(1,14)_ = 5.91, *p* = 0.029) (Fig. 8b). Moreover, we also found that all mutants made significantly more small steps and fewer large steps than control littermates (Pcd *F*_(1,10)_ = 79.79, *p* < 0.001; L7-Pp2b *F*_(1,22)_ = 118.57, *p* < 0.001; L7-∆γ2 *F*_(1,18)_ = 22.04, *p* < 0.001; α6-Cacna1a *F*_(1,14)_ = 7.76, *p* = 0.015) (Fig. 8c).

Similar to the non-perturbed sessions, the changes across the sessions during perturbed locomotion were also reflected in changes within the sessions (Suppl. Figure 2 and Suppl. Table 1). In control mice, improvement could be seen in the number of steps and missteps (e.g., first perturbed session (5): number of steps *p* = 0.001; number of missteps *p* = 0.013). Pcd mice also showed a change in their number of steps during session 5 (*p* = 0.031); yet, their overall performance remained significantly worse than that of control mice.

#### Walking pattern consistency and efficiency

During the first perturbed session (i.e., session 5), control mice changed their step length about four to five times per trial, while over the next sessions they progressively developed a steadier walking pattern (Table 1; Fig. 9a). The cerebellar mutants, except Pcd mice, were also able to decrease their step length variability as the perturbed sessions progressed (Table 1), but all groups of mutants showed walking patterns that were more inconsistent than those of the control mice (Pcd *F*_(1,10)_ = 39.69, *p* < 0.001; L7-Pp2b *F*_(1,22)_ = 48.14, *p* < 0.001; L7-∆γ2 *F*_(1,18)_ = 35.43, *p* < 0.001; α6-Cacna1a *F*_(1,14)_ = 5.48, *p* = 0.035) (Fig. 9a). Along the same line, due to the confrontation with an obstacle the mice were unable to make as many consecutive steps of the same length within the same block (i.e., block size) as they had done during non-perturbed sessions (compare Figs. 5b and 9b); both control and mutant mice were generally not able to make more than five regular steps per block (in contrast to up to 10 in the non-perturbed sessions). The Pcd, L7-Pp2b, L7-∆γ2 as well as α6-Cacna1a mice all showed a significantly increased number of consecutive small steps within the same block compared to controls (Pcd *F*_(1,10)_ = 12.13, *p* = 0.006; L7-Pp2b *F*_(1,22)_ = 45.5, *p* < 0.001; L7-∆γ2 *F*_(1,18)_ = 16.72, *p* = 0.001; α6-Cacna1a *F*_(1,14)_ = 12.9, *p* = 0.003), whereas the opposite happened with regard to large steps in Pcd, L7-Pp2b, L7-∆γ2, but not α6-Cacna1a mice (Pcd *F*_(1,10)_ = 30.94, *p* < 0.001; L7-Pp2b *F*_(1,22)_ = 64.6, *p* < 0.001; L7-∆γ2 *F*_(1,18)_ = 20.91, *p* < 0.001; α6-Cacna1a *F*_(1,14)_ = 0.7, *p* = 0.407) (Fig. 9b). Finally, all mutants showed significantly less efficient trials per session than controls (Pcd *F*_(1,10)_ = 87.33, *p* < 0.001; L7-Pp2b *F*_(1,22)_ = 141.32, *p* < 0.001; L7-∆γ2 *F*_(1,18)_ = 20.82, *p* < 0.001; α6-Cacna1a *F*_(1,14)_ = 8.81, *p* = 0.010) (Fig. 9c). As occurred during the non-perturbed sessions, the reduced efficiency of the walking pattern was more obvious in Pcd and L7-Pp2b mice than in L7-∆γ2 and α6-Cacna1a mice.

#### Temporal aspects of locomotion

The step time during perturbed sessions exhibited the same pattern as during non-perturbed sessions. Here too, cerebellar mutant mice did not differ from their control littermates in the time needed to make a single small step (step length = 2) (Fig. 10a). However, similar to non-perturbed sessions, the average time required to make a single large step (step length = 4) was compared to controls significantly longer in all the mutants, except for α6-Cacna1a mice (Pcd *F*_(1,10)_ = 14.02, *p* = 0.004; L7-Pp2b *F*_(1,22)_ = 46.31, *p* < 0.001; L7-∆γ2 *F*_(1,18)_ = 7.76, *p* = 0.012; α6-Cacna1a *F*_(1,14)_ = 1.69, *p* = 0.215) (Fig. 10a). Pcd and L7-Pp2b mice, but not L7-∆γ2 and α6-Cacna1a mice, also showed a significantly higher step time variability (i.e., CV2) than their control littermates (Pcd *F*_(1,10)_ = 13.15, *p* = 0.005; L7-Pp2b *F*_(1,22)_ = 26.90, *p* < 0.001; L7-∆γ2 *F*_(1,18)_ = 1.48, *p* = 0.239; α6-Cacana1a *F*_(1,14)_ = 3.30, *p* = 0.091) (Fig. 10b). Interestingly, except for the L7-∆γ2 and virtually all control groups, the mice were not able to significantly reduce the variability of their step times over time (Table 1).

#### Obstacle crossing

**Fig. 11:**
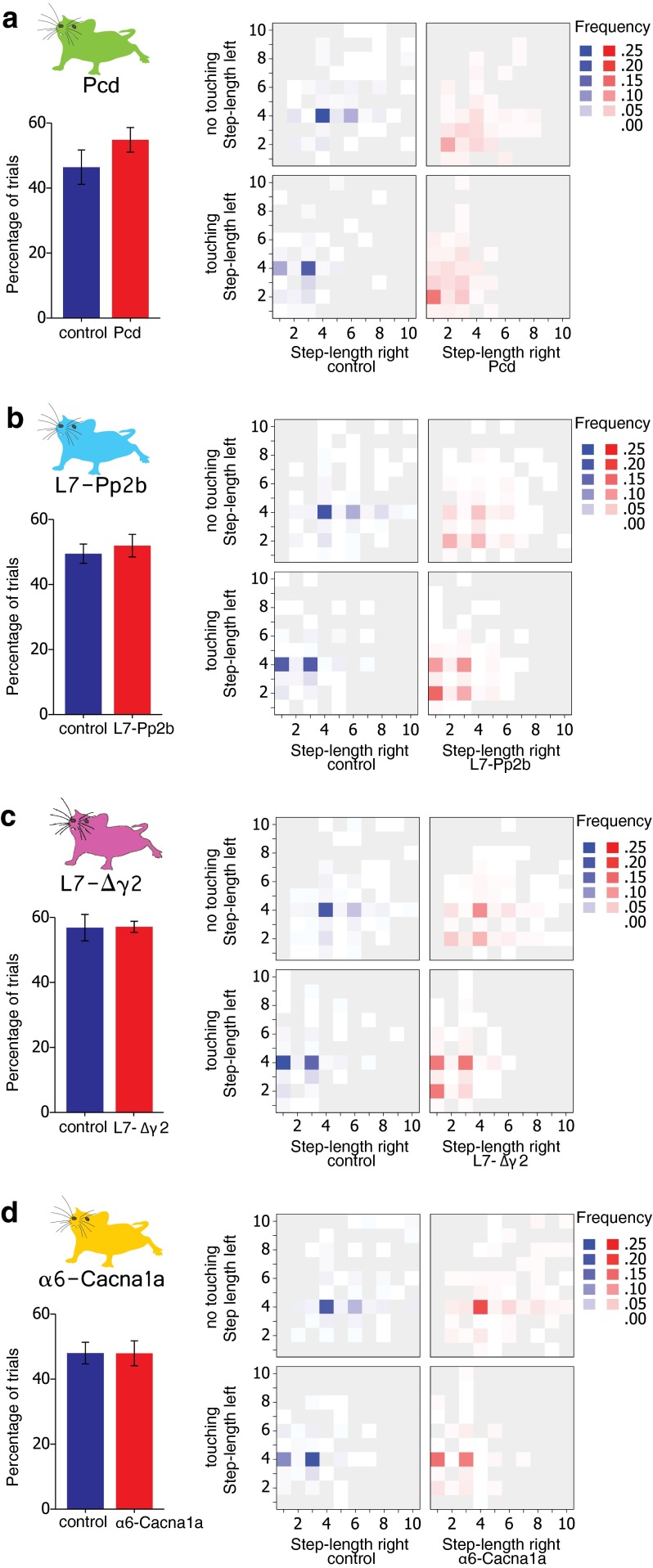
Stepping strategy during obstacle crossing. The percentage of trials in which the cerebellar mutant mice (indicated in *red*) touched the obstacle was not significantly different from that of control mice (indicated in *blue*) (*left panels*). *Panels* on the *right* show frequency distributions in which a specific step length on the side of the obstacle (*right*; *x* axis) occurred concomitantly with a specific step length on the *left* side (*y* axis) in two situations: with (*bottom*) and without (*top*) touching the obstacle. When the obstacle was not touched, control mice made large steps (step length = 4) or leaps (step length >4) on both sides. In contrast, when touching the obstacle, they combined large steps with irregular steps (either step length = 1 or 3). **a** Pcd mice did not show a stereotypic combination of step lengths in either situation, with or without touching the obstacle. **b** Similarly to Pcd mice, L7-Pp2b combined small steps and irregular steps on both sides, and they did not show clear combinations of step lengths. **c** L7-Δγ2 mice were able to combine large steps and leaps; however, they did this less often than control mice. **d** The α6-Cacna1a mice were almost indistinguishable from control mice

We wanted to know whether the impairments in locomotion adaptation of the mutant mice correlated with the way in which they crossed the obstacle. The obstacle can be passed by either stepping on it and thus touching it or crossing it without touching it. Neither controls nor mutants had a clear preference, since both groups made contact with the obstacle in about half of the trials (Fig. 11, left column). Indeed, the percentage of trials during which the obstacle was touched was not significantly different between mutants and controls (Pcd *F*_(1,10)_ = 1.455, *p* = 0.255; L7-Pp2b *F*_(1,22)_ = 0.539, *p* = 0.471; L7-∆γ2 *F*_(1,18)_ = 0.004, *p* = 0.953; α6-Cacna1a *F*_(1,14)_ = 0.000, *p* = 0.991) (Fig. 11). Next, we studied the actual stepping pattern in the period around the obstacle crossing. First, we examined the trials during which the mice crossed the obstacle without touching it. We correlated the length of the step on the right side (where the obstacle appeared) with that of the corresponding step on the left side. In control mice, the two most prevalent stepping patterns consisted of a large regular step (step length = 4) on the right side and an identical step on the left. The second most prevalent stepping pattern was a jump over the obstacle (step length = 6) on the right and a large regular step on the left (Fig. 11). A somewhat different situation was found during trials in which the mice stepped on the obstacle. The two most common stereotypical stepping patterns were a small or large irregular step (step length = 1 or 3, respectively) on the right side and a large regular step on the left. Together, the two “stereotypic” stepping patterns accounted for 50.6 and 39.2 % of all obstacle crossings in control mice with and without touching the obstacle, respectively. The percentage of stereotypic patterns of α6-Cacna1a mice (30.7 %) were significantly lower (*p* = 0.031, Fisher’s exact test) from that of control littermates (36.9 %) during trials in which they touched the obstacle, but not during trials in which they did not touch the obstacle (28.0 % control vs. 24.8 % α6-Cacna1a; *p* = 0.249) (Fig. 11d). The other cerebellar mutants all showed obstacle crossing patterns that were more irregular and differed from the stereotypical patterns in control mice (all *p* < 0.001; Fisher’s exact test). Taken together, our data suggest that, with a possible exception for the α6-Cacna1a mice, the cerebellar mutant mice did not systematize their stepping patterns to cross the obstacle.

### Cluster analysis

**Fig. 12:**
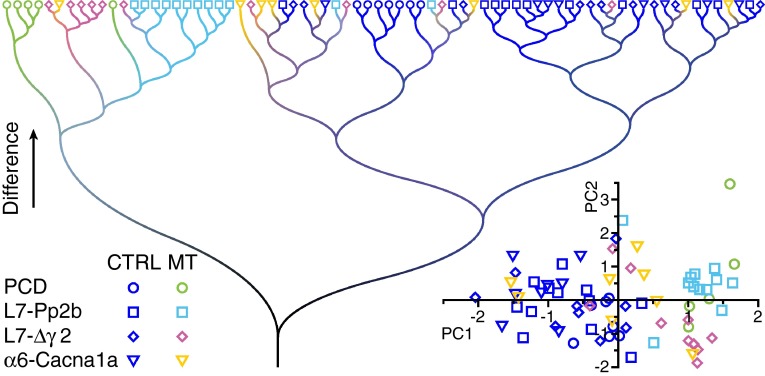
Cluster analysis reveals unique locomotor phenotypes for cerebellar mutants. In a cluster analysis on the locomotion parameters at session 5 (see “Materials and methods”) the Pcd, L7-Pp2b and L7-Δγ2 mutants form clear clusters indicating that each of them has a unique phenotype on the Erasmus Ladder. The α6-Cacna1a mice were largely interspersed between the control groups, in line with our findings that they only showed deficits at specific parameters, mostly correlated to obstacle crossing and interlimb coordination. The individual control groups were largely intermingled, indicating the absence of a systematic bias between the control groups. *Inset* Principal component analysis of the same dataset (see “Materials and methods”). The *axes* show the first two principal components (in eigenvalues). The mutant and control mice segregate largely on the first (and thus most significant) principal component (PC1, *x* axis), whereas the different mutant groups cluster apart when also the second principal component (PC2, *y* axis) is taken into account. Also in this analysis, the α6-Cacna1a mice are less different from the control groups than the other three mutant mouse *lines*

Next, we wanted to know whether the variations in locomotion patterns between the different groups of mice were larger than those observed between individual mice within these groups. To this end, we performed a principal component analysis on ten parameters of locomotion during session 5 (see “Materials and methods”). Especially, the first component revealed a good separation between mutant mice (predominantly positive eigenvalues) and control mice (predominantly negative eigenvalues) (Fig. 12, inset). Taking the first two principal components into account, we also observed a clear separation between Pcd, L7-Pp2b and L7-∆γ2 mice. Only the α6-Cacna1a mice were largely intermingled with the control mice. No obvious clustering was observed between the different groups of control mice.

These findings were further substantiated by a cluster analysis on the same ten parameters (again during session 5, see “Materials and methods”) (Fig. 12). Again, the mutant mouse lines were clearly separated from the control lines with the exception of α6-Cacna1a mice, which were not obviously different from control mice. Also in this analysis, the Pcd, L7-Pp2b and L7-∆γ2 mice largely formed their own clusters, indicating that they showed a unique phenotype on the Erasmus Ladder. As with principal component analysis, the α6-Cacna1a mice were more similar to the control groups than the other mutant mouse lines. Although the Pcd control mice tended to group together, overall the different control strains were similar to each other. Thus despite variations between individual mice, Pcd, L7-Pp2b and L7-∆γ2 mice each have a unique and clear phenotype on the Erasmus Ladder, whereas α6-Cacna1a mice show relatively normal baseline locomotion patterns and only a mild phenotype when challenged during perturbed sessions. We did not find a systematic bias between the different control groups.

### Interlimb coordination

**Fig. 13:**
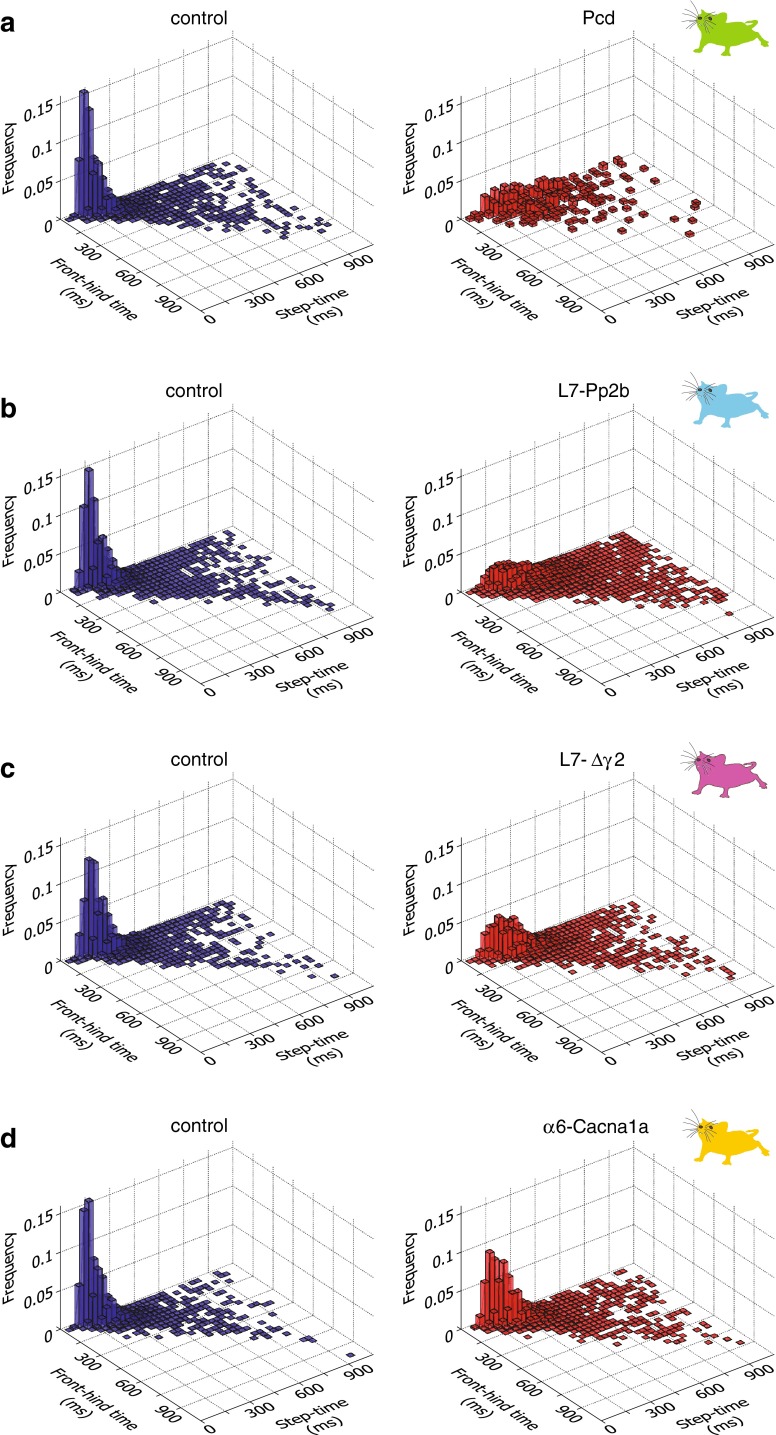
Front–hind interlimb coordination during perturbation sessions. Coordination between front and hind limbs was estimated by correlating the times between steps of front limbs and hind limbs with their respective individual step times. **a**–**d** All cerebellar mutant mice showed a much broader distribution of their front–hind times in comparison with control mice (Pcd: *p* < 0.001; L7-Pp2b: *p* < 0.001; L7-Δγ2: *p* < 0.001; α6-Cacna1a: *p* < 0.001; 2-D Kolmogorov–Smirnov test)

**Fig. 14:**
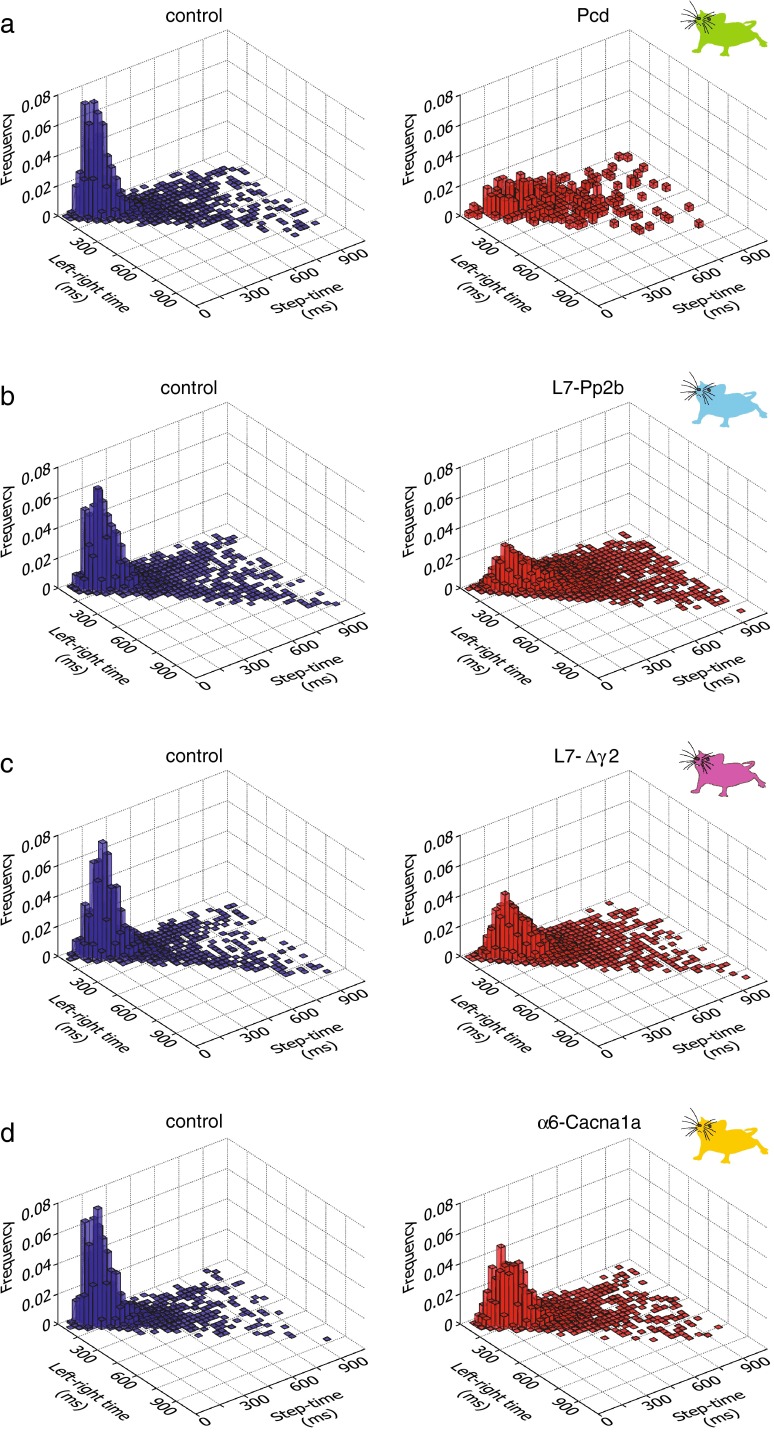
Left–right interlimb coordination during perturbation sessions. Coordination between left and right limbs was estimated by correlating the times between steps of the left and right forelimb with their respective individual step times. **a**–**d** All cerebellar mutant mice showed a much broader distribution of their left–right times in comparison with control mice (Pcd: *p* < 0.001; L7-Pp2b: *p* < 0.001; L7-Δγ2: *p* < 0.001; α6-Cacna1a *p* < 0.001; 2-D Kolmogorov–Smirnov test)

The spatial arrangement of the rungs of the ladder forced the mice to make discrete steps from one rung to the next. As a result, the hind limbs of a mouse usually followed the stepping pattern of the front limbs in that the hind paw touched the same rung previously touched by the ipsilateral front paw. All cerebellar mutant mice showed longer time intervals between front and hind limbs (“front–hind times”) than did their control littermates (all *p* < 0.001, Kolmogorov–Smirnov test). Next, we investigated front–hind limb coordination by correlating the front–hind times with the corresponding step times of the front paw (see “Materials and methods”). We found that control mice showed a regular step cycle in that their variation in front–hind times was smaller than that in mutants (Fig. 13); significant differences between controls and mutants were observed in all four genotypes during both non-perturbed (all *p* < 0.001, 2-D Kolmogorov–Smirnov test; data not shown) and perturbed sessions (all *p* < 0.001, 2-D Kolmogorov–Smirnov test) (Fig. 13; Tables 2, 3). Likewise, the interval between left and right touches was also longer in mutant than in control mice (*p* < 0.001 for all genotypes, Kolmogorov–Smirnov test). Consequently left–right coordination was also impaired in all the mutant groups (see “Materials and methods”) during both non-perturbed (all *p* < 0.001, 2-D Kolmogorov–Smirnov test; data not shown) and perturbed sessions (all *p* < 0.001, 2-D Kolmogorov–Smirnov test) (Fig. 14; Tables 2, 3). Given that both the average and variability of the step times in α6-Cacna1a mice were, just like all their other motor performance parameters during non-perturbed sessions, indistinguishable from those in their control littermates (Figs. 10, 12), it is remarkable that the variation in distribution of their front–hind as well as their left–right times was significantly higher than in controls (Figs. 13, 14). Hence, it is possible that the ultimate outcome in motor performance parameters is relatively normal, whereas the strategy toward that outcome may differ.

### Cognition

When we test mice for locomotion impairments on the Erasmus Ladder, we can also assess various cognitive parameters, such as those related to motivation and avoidance. Motivation can be tested by calculating the number of times mice react to specific stimuli meant to serve as a signal for them to leave the box. Similarly, we can test avoidance behavior by determining to what extent motivation mice are de-motivated when confronted with an aversive situation, such as an emerging obstacle. To evaluate motivation we calculated the percentage of trials per session in which the mice reacted to cues for departure, being either a friendly LED light or a more forceful air flow (see “Materials and methods”). The mice progressively began to respond to light rather than to the air flow that was switched on when they would not leave the starting shelter box on time. The number of trials during which the mice left the shelter box upon the light stimulus was interpreted as a measure of their motivation. During the perturbed sessions, the mice became more reluctant to start a trial, which was taken as a sign of avoidance behavior.

**Fig. 15:**
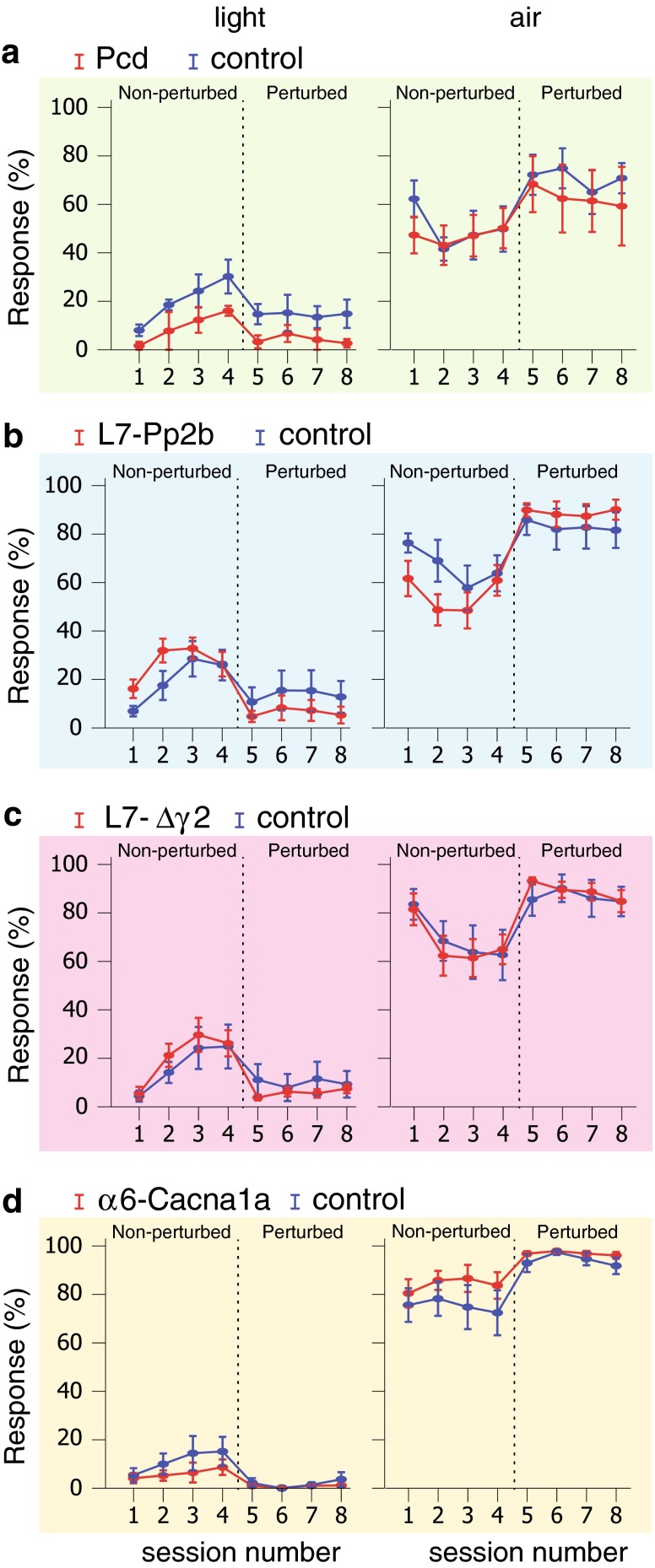
Motivation and avoidance behavior. Motivation was tested in non-perturbed sessions by calculating the percentage of trials per session in which the mice properly used the light stimulus to leave the shelter box and started to walk on the ladder. The same responses measured during unpleasant circumstances (perturbed sessions) were used to test avoidance behavior. a–d (light) The occurrence of responses to light during non-perturbed sessions was not significantly different for any of the cerebellar mutant mice from that in control mice. Moreover, the occurrence of mutant responses to light was also not significantly different from that of control mice during perturbed sessions. **a**–**d** (air) Similarly, the occurrence of responses to air stimuli in cerebellar mutant mice during non-perturbed sessions was not significantly different from that in control mice. The occurrence of responses to air was also not significantly different from that in control mice during perturbed sessions. *Error bars* represent SEM

We did not observe any clear difference either in motivation or avoidance behavior between cerebellar mutant mice and their control littermates. The percentage of trials in which Pcd, L7-Pp2b, L7-∆γ2 and α6-Cacana1a mice reacted to the light stimulus was not different from that of control littermates during either non-perturbed (Pcd *F*_(1,10)_ = 2.818, *p* = 0.124; L7-Pp2b *F*_(1,22)_ = 1.36, *p* = 0.257; L7-∆γ2 *F*_(1,18)_ = 0.28, *p* = 0.603; α6-Cacna1a *F*_(1,14)_ = 1.11, *p* = 0.309) or perturbed sessions (Pcd *F*_(1,10)_ = 3.255, *p* = 0.101; L7-Pp2b *F*_(1,22)_ = 0.81, *p* = 0.378; L7-∆γ2 *F*_(1,18)_ = 0.46, *p* = 0.504; α6-Cacna1a *F*_(1,14)_ = 0.70, *p* = 0.416) (Fig. 15, first column; Tables 2, 3). In addition, no significant difference with regard to their response to air stimuli was observed during either non-perturbed (Pcd *F*_(1,10)_ = 0.209, *p* = 0.657; L7-Pp2b *F*_(1,22)_ = 1.85, *p* = 0.187; L7-∆γ2 *F*_(1,18)_ = 0.04, *p* = 0.843; α6-Cacna1a *F*_(1,14)_ = 1.52, *p* = 0.238) or perturbed sessions (Pcd *F*_(1,10)_ = 0.371, *p* = 0.556; L7-Pp2b *F*_(1,22)_ = 0.47, *p* = 0.501; L7-∆γ2 *F*_(1,18)_ = 0.13, *p* = 0.728; α6-Cacna1a *F*_(1,14)_ = 1.41, *p* = 0.255) (Fig. 15, second column; Tables 2, 3).

## Discussion

We tested four different cerebellar mouse mutant lines, which suffer from deficient processing in their Purkinje cells, molecular layer interneurons or granule cells, on the Erasmus Ladder to study their basic walking patterns, locomotion adaptation to perturbations and interlimb coordination. Different from rotarod, open field, CatWalk or footprint analysis (Angeby-Moller et al. 2008; Galliano et al. 2013b), the Erasmus Ladder allows analyses of locomotion at all these levels. Whereas most parameters on the basic walking patterns were only affected in the mutants in which the presence and potentiation of Purkinje cells were affected (i.e., Pcd and L7-Pp2b mutants), those on locomotion adaptation and interlimb coordination were mostly affected in all four mutants (i.e., Pcd, L7-Pp2b, L7-∆γ2 and α6-Cacna1a mice), highlighting the relevance of a complete cerebellar cortical circuitry in more complicated and demanding motor tasks. In contrast, we did not observe any abnormal behavior related to cognitive parameters of motivation or avoidance.

### Developmental compensation

All four types of mouse mutants tested in the current study were suffering from cerebellar cell-specific deficits and thereby the observed phenotypes point toward the essential functions of the cellular processes affected. However, it should be noted that in all four mutants the deficits occur from early on and could potentially allow for compensatory mechanisms during development (see e.g., Wulff et al. 2009). This approach provides special opportunities in that it allows for uncovering the essential functions despite developmental compensation, but at the same time it also has its limitations in other types of questions. For example, the current approach cannot exclude the possibility that a particular cellular process does actually contribute to a particular locomotion parameter in wild-type animals, despite the fact that that particular cellular process was impaired in one of the mutants and that that particular locomotion parameter was not significantly affected in this mutant. In other words, with the current approach the presence of a phenotype is meaningful, but the absence of it has to be interpreted with caution due to issues of developmental compensation, which can obscure functional contributions that can take place under physiological circumstances without genetic deficits.

### The Erasmus Ladder

There are many experimental paradigms to characterize the locomotion pattern in small rodents. Most of these methods focus either on spatial patterns (e.g., CatWalk, footprint analysis), on general aspects of locomotion (e.g., open field test) or on balance (e.g., rotarod, balance beam). The Erasmus Ladder combines all these features and includes a precise temporal analysis of locomotion, even of four limbs independently, allowing the study of interlimb coordination. Furthermore, the mice can be challenged during perturbed sessions in which they have to cross a suddenly appearing obstacle. Thus, the Erasmus Ladder yields a more complete and quantitative analysis of locomotion than other systems currently available. With respect to particular parameters, the results obtained with the Erasmus Ladder can be comparable to those of other tests, but its precise quantification of a wide range of parameters can still reveal additional phenotypes that are hard to substantiate with more classic methods (e.g., Galliano et al. 2013a, b). One of the explanations for the sensitivity of the Erasmus Ladder may be that it forces the mice to make steps of discrete size from rung to rung requiring a relatively high level of sensorimotor integration. At the same time this feature may yield somewhat different results from other tasks, such as the CatWalk, in which mice can adjust their step size at will.

### Basic walking patterns

During non-perturbed locomotion, Pcd and L7-Pp2b mice made significantly more small steps, had a prolonged step time for large steps when they occurred and had more inconsistent stepping patterns than controls. These data emphasize the strategic and important role of Purkinje cells, which form the sole output of the cerebellar cortex. Apparently, their presence and ability to be potentiated intrinsically and/or postsynaptically at their parallel fiber synapses are critical for generating basic walking patterns. The current Erasmus Ladder data on Pcd mice complement previous descriptions of ataxic walking patterns and poor balance control in both Lurchers and Pcd mice (Fortier et al. 1987; Wang et al. 2006; Van Der Giessen et al. 2008; Cendelin et al. 2010). For example, Pcd mice have been shown to exhibit short and irregular strides recorded by footprint analysis, to have difficulties keeping balance on the rotarod and to display reduced open field locomotion activity (Triarhou et al. 1996; Wang et al. 2006). L7-Pp2b mice showed the same behavioral phenotypes as the Pcd mice, albeit quantitatively at a somewhat less prominent level. Their phenotype indicates that potentiation of Purkinje cells is more critical for baseline locomotion than LTD, as mice in which expression of LTD is blocked at the level of AMPA receptors, do not show any form of motor performance deficit during the same type of locomotion tasks on the Erasmus Ladder (Schonewille et al. 2011). So in this respect, the presence and absence of phenotypes during baseline locomotion in LTP (i.e., L7-Pp2b knockout) and LTD (i.e., PICK1 knockout, GluR2Δ7 knockin and GluR2K882A knockin) deficient mutant mice resemble those seen during compensatory eye movements or eyeblink conditioning (Schonewille et al. 2010, 2011). Since LTP at parallel fiber-to-Purkinje cell synapses and intrinsic plasticity in Purkinje cells are synergistically enhanced during the absence of climbing fiber activity (Gao et al. 2012), it is interesting to note that robust aberrations of climbing fiber innervation and/or activity can also lead to motor performance deficits. For example, mutants with severe, but not mild, persistent multiple climbing fiber innervation (e.g., Gαq mice) (Offermanns et al. 1997; De Zeeuw et al. 1998; Bosman and Konnerth 2009), mutants with aberrant laterality of their climbing fiber input (e.g., Ptf1a-Robo3 mice) (Badura et al. 2013) or mutants with strongly reduced climbing fiber activity, but intact climbing fibers (Chen et al. 2010), can all be ataxic. The dominant phenotypes seen in Pcd and L7-Pp2b mice, i.e., small steps, long-lasting step times and inconsistent patterns, resemble closely the symptoms seen in patients suffering from cerebellar ataxia in that they also show enhanced gait variability that critically depends on walking speed (Wuehr et al. 2013).

In contrast, L7-∆γ2 and α6-Cacna1a mice, which suffer from impaired input from interneurons and granule cells, respectively (Galliano et al. 2013a; Wulff et al. 2009), had few or no detectable deficits during baseline locomotion sessions on the ladder (Table 2). Apparently, no molecular layer interneurons and only a minimum number of granule cells are required to maintain baseline locomotion (i.e., when developmental compensation is allowed). In this respect the phenotypes of L7-∆γ2 and α6-Cacna1a mice on the Erasmus Ladder resemble more closely those of other mutant mice with subtle abnormalities, such as mice lacking Neuroligin-3 in Purkinje cells (Baudouin et al. 2012); mice lacking Cx36-containing gap junctions in neurons of the inferior olive (Van Der Giessen et al. 2008); or mice lacking AMPA receptors in Bergmann glia cells (Saab et al. 2012).

### Locomotion adaptation to perturbation

Some parameters, such as total number of steps, number of small steps or number of blocks, showed a significant change across the four unperturbed sessions in wild types (Table 1) and some of these learning curves were even significantly less steep in Pcd, L7-Pp2b or L7-∆γ2 mutants (Figs. 4a, c, 5a). However, these trends and differences were relatively sporadic and inconsistent across all parameters tested during the non-perturbed baseline sessions (Figs. 4, 5, 6). Instead, when we started to insert perturbations and forced the animals to adapt their walking patterns during sessions five to eight, the vast majority of all parameters showed significant differences among wild types and mutants, and this held true for all four mutants, i.e., including Pcd, L7-Pp2b, L7-∆γ2 and α6-Cacna1a mice (Figs. 8, 9, 10). Thus, in contrast to the locomotion sessions without perturbations, those with perturbations preceded by an auditory stimulus showed not only many learning curves, but also consistent and robust significant differences between wild-type littermates and mutants, independent of the cellular defect involved. The fact that all functional abnormalities translated into pronounced deficits in locomotion adaptation is in line with the phenotypes we observed in Lurcher, L7-Pp2b, L7-∆γ2 and α6-Cacna1a mice during VOR adaptation (Van Alphen et al. 2002; Wulff et al. 2009; Schonewille et al. 2010; Galliano et al. 2013a). Moreover, our finding that Pcd, L7-Pp2b and L7-∆γ2 mice also differed from controls in their strategy to cross obstacles, showing a preference for variable small step approaches, corroborates obstacle avoidance strategies in patients with cerebellar degeneration (Kim et al. 2013; Morton et al. 2004).

### Cerebellum controls interlimb coordination

All cerebellar mutants exhibited impairments in both front–hind and left–right interlimb coordination during locomotion, in that they showed more irregular step cycles than controls (Tables 2, 3). Interestingly, these impairments occurred not only in all groups of mutants during perturbed, but also during unperturbed sessions. Thus, even L7-∆γ2 and α6-Cacna1a mice, which had no significant functional deficits in number of missteps, step length, blocks, regularity and efficiency during the unperturbed sessions, still showed abnormalities in both front–hind and left–right interlimb coordination during this task. These results suggest that the coordination strategy can already be affected at all stages of the step cycle following mild cerebellar deficits, whereas the functional outcome in terms of timing and amplitude of limb movements during locomotion performance may still appear normal, rendering interlimb coordination as the most sensitive parameter for cerebellar deficits in mouse mutants. Our data are in line with cerebellar mini-lesion studies in mice exhibiting poor stride-length coupling between limbs, while leaving rotarod performance unaffected (Stroobants et al. 2013). Deficits in coordination of different muscle groups may also explain why our cerebellar mutants showed relatively robust deficits in step time of large steps (Figs. 6a, 10a), which presumably require more precise intra-limb control than smaller steps. Indeed, increased variability in both interlimb and intralimb kinematics has been recognized as a major characteristic in patients with cerebellar ataxia (Ebersbach et al. 1999; Anheim et al. 2012). Presumably, the cerebellum complements the role of the spinal cord in interlimb coordination (Zehr and Duysens 2004; Dietz 2002; Talpalar et al. 2013) by adjusting phasing between the limbs (Reisman et al. 2005; Morton and Bastian 2006).

### Cognition

None of the four types of cerebellar mutants had a deficit in their motivation to leave the box during the unperturbed sessions (Table 2) or in their tendency to avoid leaving the box during the perturbed sessions (Table 3). These outcomes indicate that the use of LED and/or puffs itself does not lead directly to behavioral phenotypes per se (Koekkoek et al. 2003; Boele et al. 2010). Moreover, the current data on the roles of specific cerebellar cell types stand in marked contrast to those obtained in other global mutants, such as the model for Fragile X (FMR1 knockout), which do show deficits in avoidance behavior (Vinueza Veloz et al. 2012). Our data are in line with another study, which showed that the L7-Pp2b, L7-∆γ2 and α6-Cacna1a mutants do not have phenotypes in cognitive tasks such as the Morris water maze, open field, social testing or fear conditioning (Galliano et al. 2013b). However, L7-Pp2b mice have severe problems in learning a whisker-based object localization task in which a narrow time-response window is engaged (Rahmati et al. 2014). We therefore hypothesize that the role of the cerebellum in cognitive tasks may be particularly prominent when precise timing in the order of tens of milliseconds is required, which was not the case in the current protocols for leaving the start and end boxes of the Erasmus Ladder.

## Electronic supplementary material

Below is the link to the electronic supplementary material.

**Supplementary Table** **1. Behavior of Pcd mice within sessions 1 to 8.** Control mice improved their performance within sessions, especially during the first non-perturbed session (session 1) and the first perturbed session (session 5). Pcd mice did not improve their performance within the non-perturbed sessions, but did show a change in the number of steps during the first two perturbed sessions. Values indicate the p values for the linear regression slope. Significant values (*p* < 0.05) are indicated in red. (EPS 742 kb)

**Supplementary Figure 1. Performance of Pcd mutant mice between and within non-perturbed sessions.** Performance of a representative control **(a)** and Pcd mutant mouse **(b)** for number of steps, number of missteps and step time CV2 during non-perturbed sessions (sessions 1 to 4). Every dot represents a single trial. **(c)** Control mice showed a trend of performance improvement within the sessions, especially during session 1 (number of steps *p* > 0.001; number of missteps *p* = 0.002; CV2 *p* = 0.001). In contrast, Pcd mice showed no improvement of their performance within sessions (number of steps *p* = 0.968; number of missteps *p* = 0.566; CV2 *p* = 0.968). (EPS 1867 kb)

**Supplementary Figure** **2. Performance of Pcd mice and control littermates between and within perturbed sessions.** Performance of a representative control **(a)** and Pcd mutant mouse **(b)** for number of steps, number of missteps and CV2 during non-perturbed sessions (session 1 to 4). Every dot represents a single trial. **(c)** In control mice performance improvement could only seen in two of the three parameters during the first perturbed session (session 5) (number of steps *p* = 0.001; number of missteps *p* = 0.013; CV2 *p* = 0.252). Interestingly, Pcd mutant mice showed some improvement in their performance during session 5 (number of steps *p* = 0.031; number of missteps *p* = 0.104; CV2 *p* = 0.954). (EPS 1923 kb)
